# Cell-specific modulation of gastrointestinal NO-induced relaxation by phosphodiesterases

**DOI:** 10.1186/2050-6511-16-S1-A56

**Published:** 2015-09-02

**Authors:** Dieter Groneberg, Annemarie Aue, Barbara Lies, Andreas Friebe

**Affiliations:** 1 Department of Physiology, University Würzburg, Würzburg, Germany

## Background

In the gastrointestinal (GI) tract NO is produced by nNOS and released from nerve varicosities to relax GI smooth muscle. NO-sensitive guanylyl cyclase (NO-GC), the receptor for NO, is expressed in at least two cell types, smooth muscle cells (SMC) and interstitial cells of Cajal (ICC). It is still unclear which of these cell types acts as primary target for nerve-released NO. ICC express much higher amounts of NO-GC than SMC and may therefore scavenge NO. In these cells, the amount of NO-GC as well as the type(s) of phosphodiesterase(s) (PDE) present will influence strength and duration of the cGMP signal. Thereby, the cGMP/cAMP crosstalk in these cells will be affected.

## Methods

To clarify the individual impact of cGMP and cAMP on relaxation of gastrointestinal smooth muscle we used our mouse lines lacking NO-GC in SMC and ICC. Isometric force studies were performed to monitor the responsiveness to exogenous and endogenous NO in absence and presence of blockers of PDE3 (milrinone) and PDE5 (sildenafil). Immunohistochemistry was employed to identify PDE3/5 expression.

## Results

PDE5 is expressed in both SMC/ICC and participates in cGMP degradation. Inhibition of PDE5 by sildenafil increases NO-induced relaxation. Surprisingly, inhibition of PDE3 expressed in SMC and ICC led to a decrease in NO-induced relaxation. We are currently investigating the impact of cGMP/cAMP crosstalk via PDE3 on GI smooth muscle relaxation.

